# Metabolomic Analysis and Identification of Sperm Freezability-Related Metabolites in Boar Seminal Plasma

**DOI:** 10.3390/ani11071939

**Published:** 2021-06-29

**Authors:** Yuting Zhang, Hanlin Liang, Yan Liu, Meng Zhao, Qianqian Xu, Zhonghua Liu, Xiaogang Weng

**Affiliations:** Key Laboratory of Animal Cellular and Genetics Engineering of Heilongjiang Province, College of Life Science, Northeast Agricultural University, Harbin 150030, China; zhangyuting217@neau.edu.cn (Y.Z.); Lhl020039@163.com (H.L.); liuyan217@neau.edu.cn (Y.L.); zm2892989908@163.com (M.Z.); xuqianqian1990@sina.com (Q.X.)

**Keywords:** pig, sperm, freezability, seminal plasma, metabolome

## Abstract

**Simple Summary:**

In the freezing process of boar sperm, there are obvious differences in freezability between individuals. Studies suggest that specific freezability markers might be useful in good (GFE) and poor freezability ejaculate (PFE) selection prior to cryopreservation. Therefore, we performed UHPLC-qTOF-MS analysis to explore the difference in the metabolic level of seminal plasma between boars with differential freezability, and the results showed that the content of D-aspartic acid, N-acetyl-L-glutamate (NAG), and inosine were significantly different. These findings present new insights into the role of metabolism in sperm freezability and provide research directions for exploring potential biomarkers of freezability.

**Abstract:**

Some potential markers of boar sperm freezability have been found in spermatozoa, but little attention has been paid to seminal plasma. The seminal plasma is composed of secretions from the testis, epididymis, and accessory sex glands. The exposure of spermatozoa to small molecules such as metabolites can affect sperm function. However, details and significance of the seminal plasma metabolome related to boar sperm freezability are unknown. Therefore, the main aim of this study was to explore the differences in the metabolic level of seminal plasma between boars with differential freezability and to explore the candidate biomarkers of semen freezability. A total of 953 metabolites were identified in boar semen plasma by UHPLC-qTOF-MS analysis, and 50 metabolites showed significant change between the GFE group and PFE group. Further, twelve metabolites were subjected to metabolic target analysis, and three metabolites (D-aspartic acid, N-acetyl-L-glutamate (NAG), and inosine) showed differences. In conclusion, there is significant difference in the metabolome of seminal plasma between GFE and PFE individuals. D-aspartic acid, NAG, and inosine in seminal plasma may be potential markers for assessing sperm cryopreservation resistance in boars.

## 1. Introduction

Artificial insemination has been widely used in pig production worldwide. However, frozen–thawed boar semen accounts for less than 1% of the semen used for insemination [1]. On one hand, boar spermatozoa in general presents low freezability because of its high cold shock sensitivity [2,3]. On the other hand, the quality of frozen-thawed boar semen shows strong variability in freezability between individuals [4]. Therefore, it is meaningful to distinguish between high and low freezability individuals before cryopreservation procedures and to select high freezability individuals for cryopreservation to improve the efficiency of artificial insemination utilizing post-thawed sperm. To solve this issue, researchers are engaged in a lot of work to distinguish good (GFE) and poor freezability ejaculates (PFE) [5,6,7].

Previous research on boar ejaculate freezability biomarkers mainly focused on proteomics. Numbers of proteins from sperm or seminal plasma, such as heat-shock protein 90 (HSP90AA1) [8], acrosin-binding protein (ACRBP) [9], triosephosphate isomerase (TPI) [9], and fibronectin 1 (FN1) [10], have been reported as markers for predicting boar ejaculate freezability [11]. In addition, a study demonstrated that genomic differences existed between good and poor freezers in the sequences of polymorphism restriction fragments of 16 candidate genetic markers [6]. Other freezability markers include patterns of sperm motile subpopulations in extended semen [12], specific kinetic parameters evaluated at the cooling step [13], and acrosin activity [14,15].

Sperm freezability is a complex phenotype and it cannot be accurately predicted based solely on conventional parameters [1,13]. Current knowledge implies that the seminal plasma is much more than a nutrient medium. Seminal plasma is composed of secretions from the testis, epididymis, and accessory sex glands. Seminal plasma contains a variety of substances, such as proteins, ions, and metabolites including amino acids, lipids, nucleosides, minerals, electrolytes, and steroid hormones [16,17]. As metabolites are the final products of metabolism, changes in their composition and content can reflect the state of the sperm and individual metabolic timeliness [18]. A recent study shows that metabolites play a role in sperm energy production, motility, pH control, and regulation of metabolic activity [19]. Furthermore, metabolites in seminal plasma may affect downstream and complementary changes in gene/protein expression [20]. Thus, we hypothesized that particular metabolites in seminal plasma could be considered as markers for sperm freezability.

Therefore, the aims of this study were to compare the metabolome of seminal plasma between GFE and PFE as well as identifying potential metabolites as biomarkers of freezability. We used an Ultra-high Performance Liquid Chromatography-Quadrupole Time-of-Flight Mass Spectrometry (UHPLC-qTOF-MS) based metabolomics approach to obtain the metabolic profile of seminal plasma from boars with good and poor sperm freezability. Furthermore, the potential metabolites were confirmed by targeted metabolomics analysis. Findings in the present study will provide a new perspective for boar sperm freezability prediction.

## 2. Materials and Methods

### 2.1. Sample Collection and Preparation of Seminal Plasma

The boars (*n* = 10) were chosen based on production records over 2 years. All boars were Landrace, and they were raised under the same management conditions and received the same nutrition. Semen was collected using the gloved hand method. The semen collection rhythm was twice a week, and one single ejaculate per boar was used in this study. 

After collection, the spermatozoa-rich fraction of each ejaculate (80–100 mL) was filtered through gauze and subsequently divided into two aliquots of equal volume. The first one was used for seminal plasma separation from spermatozoa through centrifugation at 500× *g* and 4 °C for 30 min. Seminal plasma preparations were then examined using phase microscopy to ensure no spermatozoa remained. Clean seminal plasma samples were then stored in liquid nitrogen. Another spermatozoa-rich fraction aliquot was diluted in Androhep Plus (Minitube co., ltd., Hauptstrasse, Germany) at 2 × 10 [8] and then used to cryopreserve.

### 2.2. Cryopreservation and Thawing of Sperm Samples

Firstly, the semen samples were stored at 17 °C to cool, then centrifuged at 500× *g* for 10 min. Soft sperm pellets were subsequently diluted to 2 × 10 [9] spermatozoa/mL in Androstar Cryo Plus (Minitube, Germany) containing 20% egg yolk. Then, the spermatozoa were cooled slowly to 5 °C for 5 h and subsequently diluted to 1 × 10 [9] spermatozoa/mL with a freezing medium containing 6% glycerol (Sigma-Aldrich, St. Louis, MO, USA) at 5 °C. Afterward, sperm samples were packed in 0.5 mL labeled plastic straws (Minitube, Germany). The straws were then transferred to a programmable freezer (CryoMed 7457 (Thermo Fisher, Waltham, MA USA)). The cooling ramp was as follows: wait at 4 °C → 2 °C/min to 2 °C → hold for 1 min at 2 °C → 35 °C/min to −30 °C → hold for 1 min at −30 °C → 35 °C/min to −150 °C → hold for 4 min at −150 °C. The straws were finally plunged into liquid nitrogen and stored before use.

### 2.3. Assessment of Sperm Quality

Sperm motility parameters obtained were those described by Yeste et al. [21]. Sperm motility assessment was carried out utilizing a commercial computer assisted sperm analysis (CASA) system (CASAS-QH-III, Tsinghua Tongfang Co., Ltd., Beijing, China). After evaluating three replicates per sample (a minimum of 1000 spermatozoa were counted per replicate), the corresponding mean standard error of the mean (SEM) was calculated.

### 2.4. Sample Classification into GFEs and PFEs

To classify seminal plasma samples into two groups (GFEs vs. PFEs), spermatozoa were cryopreserved and thawed; sperm quality assessments were carried out at three different points: pre-freeze, refrigerated semen at 17 °C, and frozen–thawed spermatozoa at 30 min post thawing. To distinguish seminal plasma samples between two groups of good (GFE) and poor (PFE) freezability, boar sperm was characterized by a reduced sperm motility.

### 2.5. Ultra-High Performance Liquid Chromatography-Quadrupole Time-of-Flight Mass Spectrometry (UHPLC-qTOF-MS) Data Acquisition

Analysis data were acquired using a UHPLC-high definition quadrupole time-of-flight MS instrument (UHPLC-qTOF SYNAPT G1 HD-MS system, Waters Co., Ltd., Milford, MA, USA), equipped with a TripleTOF 6600 (Q-TOF, AB Sciex). A binary solvent method consisting of eluent A (25 mM NH4Ac and 25 mM NH4OH in water pH = 9.75) and acetonitrile (B) was carried out with an elution gradient as follows: 0 min, 95% B; 0.5 min, 95% B; 7 min, 65% B; 8 min, 40% B; 9 min, 40% B; 9.1 min, 95% B; 12 min, 95% B, delivered at 0.5 mL min^−1^. The Triple TOF mass spectrometer was used for its ability to acquire MS/MS spectra on an information-dependent basis (IDA) during an LC/MS experiment. In this mode, the acquisition software (Analyst TF 1.7, AB Sciex) continuously evaluated the full-scan survey MS data as it collected and triggered the acquisition of MS/MS spectra depending on preselected criteria. In each cycle, 12 precursor ions, whose intensity was greater than 100, were chosen for fragmentation at a collision energy (CE) of 30 V (15 MS/MS events with product ion accumulation time of 50 ms each). ESI source conditions were set as follows: ion source gas 1 as 60 psi, ion source gas 2 as 60 psi, curtain gas as 35 psi, source temperature 600 °C, Ion Spray Voltage Floating (ISVF) 5000 V or −4000 V in positive or negative modes, respectively.

### 2.6. Multivariate Data (MVD) Analysis

UHPLC-qTOF-MS data were analyzed using SIMCA 13 software (Umetrics, Umea, Sweden) and interactive XCMS (version 3.2). Before exporting the data to SIMCA for visualization and biomarker selection, the LC-MS raw data were first processed (noise elimination, peak picking, alignment, and retention time correction) with MarkerLynxTM software (version 4.1, Waters Corporation, Milford, MA, USA). The following parameters were used for data processing: retention time (Rt) range of 2.5–11 min, mass range of 100–1000 Da, mass tolerance of 0.02 Da, and an Rt window of 0.2 min. The data matrix obtained from MarkerLynxTM processing was then exported into SIMCA 13 for PCA and OPLS-DA analysis. The data were Pareto-scaled, and no transformation was used. For the XCMS analysis, the MassLynxTM raw data (.raw) were converted to NetCDF format using the DataBridge application in MassLynxTM (Waters, Co., Ltd., Milford, MA, USA). The converted data (NetCDF format) were then used in XCMS for processing, statistical analysis, visualization, and biomarker identification as described by Chang et al. [22]. The parameters were as follows: feature detection set as centWave method, minimum peak width = 5, maximum peak width = 20, retention time correction set as Obiwarp method, Profstep = 1, alignment set as m/z width = 0.015, min fraction = 0.5, and bw = 5; statistics were set as statistical test = unpaired parametric *t*-test (Welch *t*-test), paired *t*-test, and post hoc analysis with the threshold *p*-value = 0.01 and fold-change = 1.5.

### 2.7. Relative Distribution and Statistical Analysis

Total intensity values (integrated area under the peak) from MarkerLynxTM XS software (Waters Corporation, Manchester, UK) pre-processed data matrixes were used for univariate statistical analyses. SPSS software (IBM SPSS Statistics for Windows, Version 22., Armonk, NY, USA; IBM Inc., Chicago, IL, USA) was used for such descriptive statistics. Here, Univariate Analysis of Variance (ANOVA) was performed as two-tailed complete randomized blocks and used to compare the non-treated with the different time points of treated cells. ANOVA was followed by the Bonferroni post hoc test where differences between the means were considered significant at *p* < 0.05 and indicated in the box-and-whiskers plots.

### 2.8. Targeted Metabolomics Analysis

Targeted metabolomics analysis was performed using QTRAP 5500 (AB SCIEX). The target metabolomics metabolite extraction method is the same as that of UHPLC-qTOF-MS data acquisition. We performed absolute quantification of candidate differential metabolites based on standard products, and the standard products were purchased from Yuanye Biological Technology Co., Ltd. (Shanghai, China).

## 3. Results

### 3.1. Classification of Boar Ejaculates into GFE and PFE Groups

**Figure 1:**
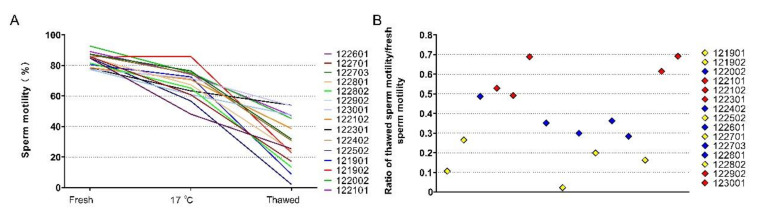
Screening semen for differential freezability. (**A**) sperm motility during freezing and after thawed, (**B**) relative sperm motility, ratio of thawed sperm motility/fresh sperm motility.

The semen collected from the selected boars showed similar sperm motility. Fresh sperm with motility higher than 75% was processed for freezing. The sperm motility of fresh sperm, kept at 17 °C and then thawed, was analyzed (Figure 1A). The difference in the sperm freezability of these boars was evaluated based on the ratio of thawed motility to fresh motility (the relative sperm motility) (Figure 1B). Five GFE and five PFE semen were chosen.

### 3.2. Metabolomic Analysis Based on UHPLC-qTOF-MS Technology

**Figure 2:**
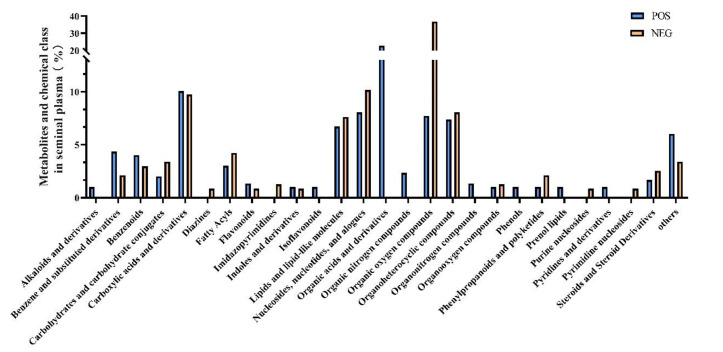
Metabolites and chemical class of seminal plasma.

A total of 953 metabolites were identified after UHPLC-qTOF-MS analysis of these seminal plasma samples, regardless of group. Metabolites were identified and categorized according to their major chemical classes, including carboxylic acids and derivatives, organooxygen compounds, amino acids, peptides, analogues, fatty amides, fatty acyls, benzene and substituted derivatives, purine nucleotides, pyrimidine nucleotides, glycosyl compounds, fatty acids, and conjugates (Figure 2). A total of 534 (POS, 298; NEG, 236) features could be mapped to current databases. According to the classification of metabolites, it was found that in the POS mode the main metabolites were organic acids and their derivatives, which contains 68 metabolites, accounting for 24% of all metabolites detected. The remaining metabolites were carboxylic acids and their derivatives (10%); nucleosides, nucleotides, and analogues ranked third (8%); followed by the organic oxygen compounds, organoheterocyclic compounds, lipids and lipid-like molecules, benzene and its substituted derivatives, benzenoids, and the other 14 metabolites (Figure 2). In the NEG mode, 87 kinds of organic oxygen compound metabolites were detected, accounting for 37% of all metabolites detected, followed by nucleosides, nucleotides, and analogues (10%), carboxylic acids and their derivatives (10%) ranked third, followed by organoheterocyclic compounds (8%), lipids and lipid-like molecules (8%), fatty acyls (4%), and 12 other metabolites (Figure 2).

### 3.3. Identification of Potential Freezability Biomarkers

**Figure 3:**
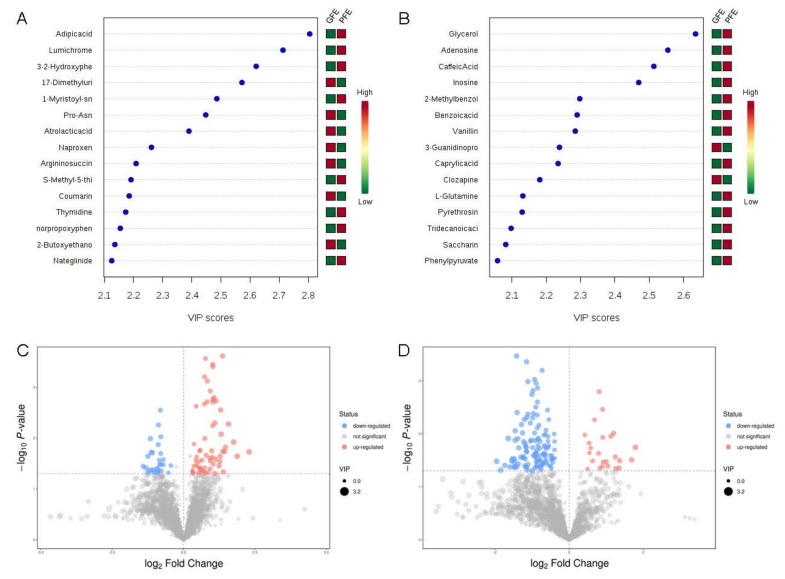
VIP value and volcano maps of metabolites. VIP scores of metabolites in GFE and PFE (TOP15) obtained in positive (**A**) and negative mode (**B**). The heat map with red or green boxes on the right indicates a high and low abundance ratio, respectively, of the corresponding metabolite in GFE and PFE. Volcano maps of metabolites in the positive (**C**) and negative (**D**) mode.

To identify potential biomarkers in seminal plasma associated with sperm freezability, PCA and OPLS-DA models were applied to the classification of the GFE and PFE groups. The quality of the OPLS-DA model was checked by seven-fold cross-validation (Figure S1). The model’s information was shown in Table S1. The two groups of samples have not been processed in any way, so the PCA analysis has not been clearly distinguished, which does not affect the subsequent analysis (Figure S1A,B). There was a clear separation between the two groups in both positive (Figure S1C) and negative ion mode (Figure S1D) in the OPLS-DA score plot. We shuffled groups in permutation tests to construct the OPLS-DA model randomly, as results show that the overall R2Y and Q2 of the original model are higher than the R2Y and Q2 of the model constructed by the replacement test, indicating that the original model grouping is not over-fitting (Figure 3E,F).

**Figure 4:**
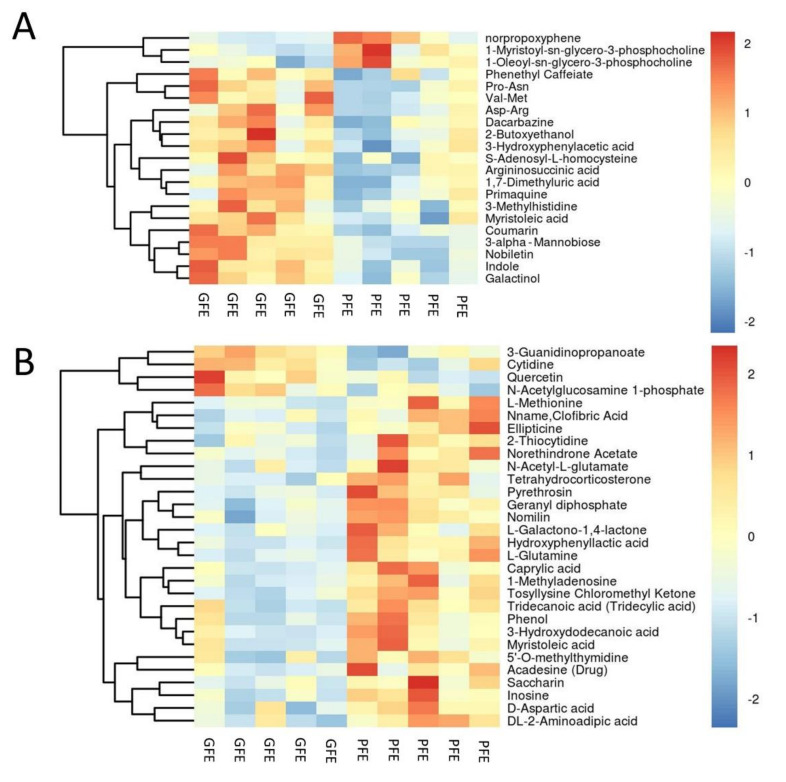
Heat map of metabolites with different content between the GFE and PFE groups. (**A**,**B**) was analyzed in positive and negative ions mode, respectively.

Based on the analysis of the OPLS-DA method, the calculated VIP and *p* value is shown in Figure 3. According to the OPLS-DA and volcano plot, metabolites with a VIP score greater than 1 and a *p*-value less than 0.05 were identified and considered as candidate freezability markers. The cluster analysis of each candidate metabolite is shown in Figure 4A,B. Finally, a total of 50 metabolites showed significant differences between the GFE and PFE groups (Tables S2 and S3).

### 3.4. Pathway Analysis of Metabolites

**Figure 5:**
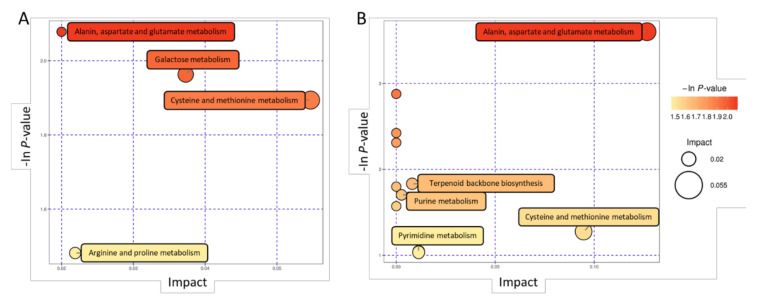
Metabolic pathway analysis of changed metabolites identified in boar seminal plasma. (**A**,**B**) was analyzed in positive and negative ions modes, respectively.

We analyzed the differential metabolic pathways via KEGG analysis (Table S4). These differential metabolites were mainly enriched in amino acid biosynthetic metabolic pathways such as alanine, aspartic acid, glutamic acid, arginine, proline, cysteine, and methionine biosynthetic metabolic pathways. Some of the metabolites are enriched in purine metabolism, pyrimidine metabolism, terpenoid backbone biosynthesis, aminoacyl tRNA biosynthesis, and other metabolic pathways (Figure 5A,B).

### 3.5. Confirmation of Freezability Biomarkers by Targeted Metabolic Analysis

**Figure 6:**
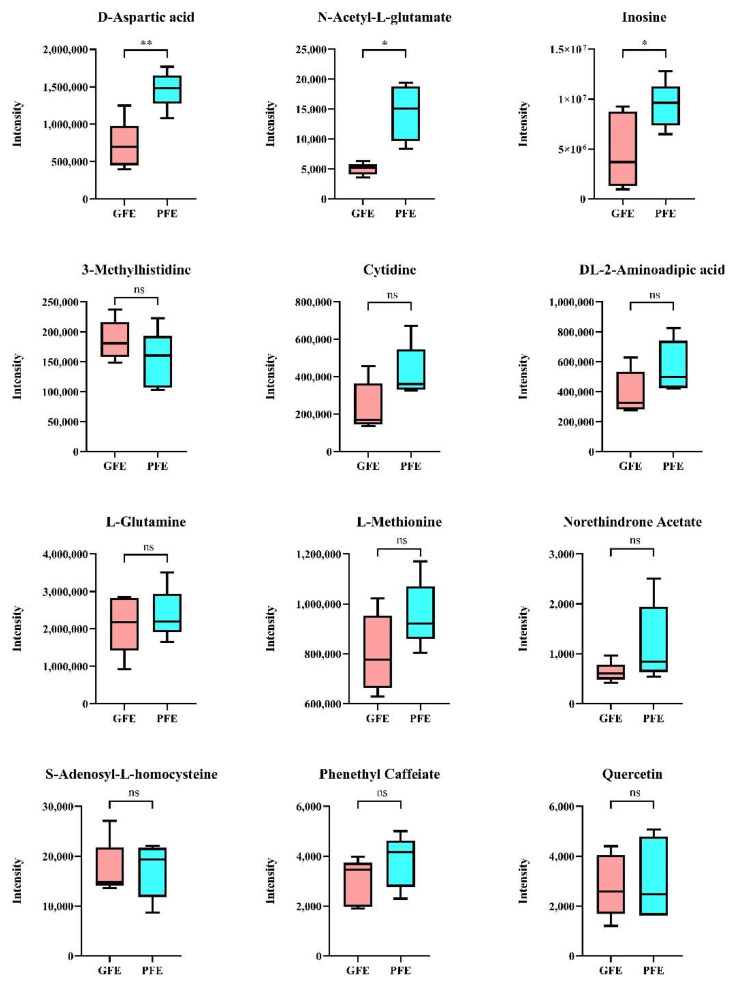
Content of metabolites confirmed by targeted metabolic analysis. Asterisk denotes significant differences between GFE within PFE. * *p* < 0.05; ** *p* < 0.01.

Among the 50 candidate metabolites obtained from the above analysis, 3-methylhistidine (*p* = 0.036), phenethyl caffeate (*p* = 0.049), S-adenosyl-L-homocysteine (*p* = 0.049), D-aspartic acid (*p* = 0.031), L-methionine (*p* = 0.018), DL-2-aminoadipic acid (*p* = 0.014), L-glutamine (*p* = 0.009), N-acetyl-L-glutamate (NAG) (*p* = 0.009), cytidine (*p* = 0.023), inosine (*p* = 0.012), quercetin (*p* = 0.034), and norethindrone acetate (*p* = 0.024) were chosen and further verified by targeted metabolism. The result reveals that D-aspartic acid, NAG, and inosine showed significant differences between GFE and PFE (*p* < 0.05) (Figure 6).

## 4. Discussion

Cryopreservation of sperm is important for the preservation of the boar sperm. In general, 40–50% of the sperm population cannot survive after cryopreservation, even when “optimized” cooling/thawing protocols are used [1,23,24]. There is a considerable variability between ejaculates in their ability to withstand cryopreservation procedures. Mammalian seminal plasma is mainly formed by secretions of the epididymis and accessory sex glands [25]. Seminal plasma contains large spectra of metabolites; the current concept states that seminal plasma can modulate sperm function. Previously, metabolites have been identified in bull and boar seminal plasma, and attempts were made to explore the candidate biomarkers of fertility [26]. The current study aimed to find a specific metabolomics signature in the seminal plasma of high freezability boars. Our study is in fact the first to compare the seminal plasma metabolome of boars between GFE and PFE. This model gives a global view of the metabolites in boar seminal plasma with both high and low freezability. Moreover, we confirmed the candidate metabolite biomarkers by utilizing the targeted metabolome method. In general, the main compounds in boar seminal plasma in the present study were carboxylic acids and derivatives, organonitrogen compounds, amino acids, peptides and their analogues, fatty acyls, purine nucleosides, pyrimidine nucleosides, and fatty acids and their conjugates. The changed 50 metabolites were enriched in amino acid biosynthetic metabolic pathways, purine metabolism, pyrimidine metabolism, terpenoid backbone biosynthesis, aminoacyl tRNA biosynthesis, and other metabolic pathways. The results implied that amino acid metabolism plays an important role in the regulation of freezability. Based on functional analysis such as KEGG, we chose 12 metabolites that could be confirmed by targeted metabolic analysis. Finally, the results confirm that D-aspartic acid, NAG, and inosine were lower in the GFE group than the PFE group. 

It is interesting that the level of D-aspartic acid was higher in the GFE group than the PFE group. Previous studies reported that D-aspartic acid occurs in human seminal plasma, and the concentration of D-aspartic acid was significantly reduced in oligoasthenoteratospermic individuals [27]. Then, studies on Leydig cells and spermatogonia in vitro demonstrated a direct effect of D-aspartic on the steroidogenic pathway and spermatogenesis. Therefore, D-aspartic mainly functions as a modulator of spermatogenesis in mammals [28]. Further, D-aspartic treatment can increase the motility of sperm [29]. However, attempts at using D-aspartic to improve the reproductive activity in animals of commercial interest have yielded mixed results. The higher concentration of D-aspartic acid in seminal plasma might impede the sperm cryotolerance ability. 

NAG is synthesized from acetyl-CoA and glutamate by N-acetyl glutamate kinase, which catalyzes the key regulatory step in the pathway to arginine biosynthesis. Moreover, in mammals, NAG is an allosteric catalyst of carbamoyl phosphate synthase-I (CPS-I) [30]. Carbamoyl phosphate and ornithine are catalyzed by CPS-I to produce citrulline [31]. Therefore, the NAG is the essential co-factor of CPS1 in the urea cycle [32]. Mammalian NAG is found in the mitochondrial matrix of cells in the liver and intestines [33]. The lower NAG level might result from the low enzyme activity of N-acetyl glutamate kinase. There was a study that reported the existence of ionotropic glutamate receptors and glutamate transporters in sperm [34]. The study also indicated that glutamate receptors and transporters might have functions other than neurotransmission in sperm [34]. The different level of NAG between the GFE and PFE groups implies that the amino acid biosynthesis is related to sperm freezability. 

Inosine is the main substance in the pathway of uric acid metabolism. Inosine has good permeability to the cell membrane and can directly enter the cell, convert it into nucleotides, and then further become ATP to participate in metabolism [35]. Exogenous inosine could accelerate the differentiation of rat intestinal epithelial cells [36]. However, there are only few studies reporting inosine in seminal plasma [37]. It seems that the inosine level was significantly higher in the seminal plasma of oligozoospermic and azoospermic than normozoospermic men [35]. Inosine in seminal plasma might activate pyruvate oxidases, increase the activity of coenzyme A, and stimulate metabolism in sperm. The negative correlation between inosine levels and sperm freezability indicates the role of nucleosides metabolism in sperm cold shock sensitivity. 

In the targeted metabolic analysis, some metabolites showed no significant differences between the two groups. However, they might also affect other functions of sperm. For example, 3-methylhistidine in urine has been shown to be correlated with protein catabolism in skeletal muscle [38]. It can be speculated that 3-methylhistidine in semen plasma can indicate the body’s metabolic state and sperm function. The functions of these metabolites in semen plasma and sperm need to be further analyzed in the future.

In summary, for the first time, we found that there are significant differences in metabolomic profiles between GFE and PFE individuals. Furthermore, some candidate metabolites were confirmed by the targeted metabolic analysis. It can be inferred that one indicator alone may not be able to accurately evaluate and that multiple markers may be needed to predict sperm freezability. It will be meaningful to evaluate sperm freezability in combination with genome, proteome, metabolome, and epigenome data.

## 5. Conclusions

This study, for the first time, investigated the metabolome profile of boar seminal plasma with high and low freezability. Fifty metabolites show significant difference between the GFE and PFE groups. The carboxylic acids and derivatives, amino acid, peptides and analogues, organooxygen compounds, and fatty amides are the main components of these changed metabolites. Moreover, our results indicate that D-aspartic acid, NAG, and inosine might be the potential markers associated with freezability. Further studies would be required to investigate the mechanism underlying the relationship between metabolites and sperm freezability. 

## Data Availability

The datasets during and/or analyzed during the current study are available from the corresponding author on reasonable request.

## Supplementary Materials

The following are available online at https://www.mdpi.com/article/10.3390/ani11071939/s1, Table S1. Model’S information, Table S2. Candidate differential metabolites screened in positive mode, Table S3. Candidate differential metabolites screened in negative mode, Table S4. KEGG pathways analysis of the differential metabolites pathway.
