# Supplementary material for: Metabolomic Analysis and Identification of Sperm Freezability-Related Metabolites in Boar Seminal Plasma
Source: Animals (Basel). 2021 Jun 29;11(7):1939. doi: 10.3390/ani11071939 (PMC8300243; doi:10.3390/ani11071939)
Supplement: Supplementary file 1 [file animals-11-01939-s001.zip › animals-1212539-supplementary.pdf]

Table S1 Model'S information

Type	R2X(cum)	R2Y(cum)	Q2(cum)
PCA (POS)	0.523		
PCA (NEG)	0.599		
OPLS-DA (POS)	0.523	0.945	0.267
OPLS-DA (NEG)	0.504	0.951	0.455

Table S2 Candidate differential metabolites screened in positive mode

MS2 name	MEAN GFE	MEAN PFE	VIP	P-VALUE	FOLD CHANGE
1,7-Dimethyluric acid	0.0942	0.0379	2.5269	0.0083	2.4825
1-Myristoyl-sn-glycero-3-phosphocholine	0.0052	0.0137	2.1016	0.0359	0.3759
1-Oleoyl-sn-glycero-3-phosphocholine	0.0016	0.0028	1.7158	0.0272	0.5566
2-Butoxyethanol	0.0259	0.0122	1.9992	0.0496	2.1198
3.alpha.-Mannobiose	4.1080	2.3077	1.8162	0.0008	1.7801
3-Hydroxyphenylacetic acid	0.1205	0.0723	1.5893	0.0458	1.6674
3-Methylhistidine	1.1480	0.8328	1.1579	0.0356	1.3786
Argininosuccinic acid	0.0390	0.0179	2.3860	0.0262	2.1754
Asp-Arg	0.0749	0.0378	2.0141	0.0178	1.9786
Coumarin	0.0176	0.0084	2.0150	0.0016	2.1020
Dacarbazine	0.0775	0.0428	1.6797	0.0476	1.8089
Galactinol	0.5637	0.3181	1.6433	0.0019	1.7721
Indole	0.0433	0.0262	1.5523	0.0021	1.6541
Myristoleic acid	0.0162	0.0118	1.1880	0.0233	1.3761
Nobiletin	0.0272	0.0164	1.7471	0.0006	1.6621
norpropoxyphene	0.0028	0.0069	2.2637	0.0386	0.4030
Phenethyl Caffeiate	0.0219	0.0131	1.4684	0.0494	1.6794
Primaquine	1.3228	1.0533	1.1033	0.0467	1.2558
Pro-Asn	0.0040	0.0017	2.3885	0.0229	2.4268

Table S3 Candidate differential metabolites screened in negative mode

MS2 name	MEAN GFE	MEAN PFE	VIP	P-VALUE	FOLD CHANGE
Tridecanoic acid (Tridecylic acid)	0.2783	0.6348	2.1164	0.0153	0.4384
Tosyllysine Chloromethyl Ketone	0.0120	0.0199	1.6392	0.0006	0.6027
Tetrahydrocorticosterone	0.0206	0.0426	1.4649	0.0138	0.4821
Saccharin	0.1809	0.5057	2.4826	0.0422	0.3577
Quercetin	0.5422	0.2871	1.6347	0.0343	1.8883
Pyrethrosin	0.0056	0.0154	2.0506	0.0298	0.3597
Phenol	0.0525	0.0866	1.4885	0.0362	0.6054
Norethindrone Acetate	0.1871	0.3038	1.4178	0.0241	0.6158
Nomilin	4.0172	5.8818	1.3541	0.0054	0.6830
Nname,Clofibric Acid	0.0258	0.0524	1.8773	0.0131	0.4927
N-Acetyl-L-glutamate	0.0198	0.0346	1.5573	0.0320	0.5740

N-Acetylglucosamine 1-phosphate	0.1879	0.1014	1.5174	0.0417	1.8531
Myristoleic acid	0.3421	0.7712	1.8389	0.0427	0.4436
L-Methionine	0.1183	0.2343	1.7950	0.0182	0.5052
L-Glutamine	0.0513	0.1409	2.2264	0.0088	0.3640
L-Galactono-1,4-lactone	0.0082	0.0202	1.9233	0.0276	0.4045
Inosine	7.1245	22.2369	2.6884	0.0123	0.3204
Hydroxyphenyllactic acid	0.0556	0.1204	1.9547	0.0010	0.4615
Geranyl diphosphate	0.0421	0.0768	1.7488	0.0044	0.5485
Ellipticine	0.1565	0.2469	1.3680	0.0177	0.6337
DL-2-Aminoadipic acid	0.2106	0.2768	1.1342	0.0140	0.7611
D-Aspartic acid	0.0685	0.0959	1.1970	0.0310	0.7142
Cytidine	0.1239	0.0715	1.5283	0.0234	1.7324
Caprylic acid	0.5436	1.4953	2.2286	0.0132	0.3635
Acadesine (Drug)	0.0146	0.0336	1.7311	0.0400	0.4358
5'-O-methylthymidine	0.0151	0.0303	2.0358	0.0498	0.4992
3-Hydroxydodecanoic acid	0.0328	0.0745	1.7901	0.0298	0.4403
3-Guanidinopropanoate	0.2673	0.1166	1.9724	0.0098	2.2931
2-Thiocytidine	0.5193	0.9476	1.5898	0.0415	0.5480
1-Methyladenosine	0.0051	0.0090	1.7015	0.0122	0.5626

Table S4 KEGG pathways analysis of the differential metabolites pathway

Pathway	model
Metabolic pathways - Sus scrofa (pig)	POS
Biosynthesis of amino acids - Sus scrofa (pig)	POS
Caffeine metabolism - Sus scrofa (pig)	POS
Galactose metabolism - Sus scrofa (pig)	POS
Histidine metabolism - Sus scrofa (pig)	POS
Phenylalanine metabolism - Sus scrofa (pig)	POS
Phenylalanine, tyrosine and tryptophan biosynthesis - Sus scrofa (pig)	POS
Tryptophan metabolism - Sus scrofa (pig)	POS
2-Oxocarboxylic acid metabolism - Sus scrofa (pig)	NEG
ABC transporters - Sus scrofa (pig)	NEG
Aminoacyl-tRNA biosynthesis - Sus scrofa (pig)	NEG
AMPK signaling pathway - Sus scrofa (pig)	NEG
Antifolate resistance - Sus scrofa (pig)	NEG
Ascorbate and aldarate metabolism - Sus scrofa (pig)	NEG
Biosynthesis of amino acids - Sus scrofa (pig)	NEG
Central carbon metabolism in cancer - Sus scrofa (pig)	NEG
D-Glutamine and D-glutamate metabolism - Sus scrofa (pig)	NEG
Fatty acid biosynthesis - Sus scrofa (pig)	NEG
GABAergic synapse - Sus scrofa (pig)	NEG
Glutamatergic synapse - Sus scrofa (pig)	NEG
Glyoxylate and dicarboxylate metabolism - Sus scrofa (pig)	NEG
Lysine degradation - Sus scrofa (pig)	NEG
Metabolic pathways - Sus scrofa (pig)	NEG
Mineral absorption - Sus scrofa (pig)	NEG
Nitrogen metabolism - Sus scrofa (pig)	NEG
Proximal tubule bicarbonate reclamation - Sus scrofa (pig)	NEG
Purine metabolism - Sus scrofa (pig)	NEG
Pyrimidine metabolism - Sus scrofa (pig)	NEG
Terpenoid backbone biosynthesis - Sus scrofa (pig)	NEG
Ubiquinone and other terpenoid-quinone biosynthesis - Sus scrofa (pig)	NEG

Alanine, aspartate and glutamate metabolism - Sus scrofa (pig)	both
Arginine biosynthesis - Sus scrofa (pig)	both
Cysteine and methionine metabolism - Sus scrofa (pig)	both
Protein digestion and absorption - Sus scrofa (pig)	both
Tyrosine metabolism - Sus scrofa (pig)	both
